# Identification of key genes in calcific aortic valve disease by integrated bioinformatics analysis

**DOI:** 10.1097/MD.0000000000021286

**Published:** 2020-07-17

**Authors:** Peng Teng, Xingjie Xu, Chengyao Ni, Haimeng Yan, Qianhui Sun, Enfan Zhang, Yiming Ni

**Affiliations:** a Department of Cardiothoracic Surgery; b Department of Bone Marrow Transplantation Center; c Department of Surgical Intensive Care Unit, The First Affiliated Hospital, College of Medicine, Zhejiang University, Hangzhou, Zhejiang Province, P.R. China.

**Keywords:** bioinformatics analysis, calcific aortic valve disease, hub genes, immune response, inflammatory response

## Abstract

Calcific aortic valve disease (CAVD) is highly prevalent in our aging world and has no effective pharmaceutical treatment. Intense efforts have been made but the underlying molecular mechanisms of CAVD are still unclear.

This study was designed to identify the critical genes and pathways in CAVD by bioinformatics analysis. Microarray datasets of GSE12644, GSE51472, and GSE83453 were obtained from Gene Expression Omnibus database. Differentially expressed genes (DEGs) were identified and functional and pathway enrichment analysis was performed. Subsequently, the protein–protein interaction network (PPI) was constructed with Search Tool for the Retrieval of Interacting Genes and was visualized with Cytoscape to identify the most significant module. Hub genes were identified by Cytoscape plugin cytoHubba.

A total of 179 DEGs, including 101 upregulated genes and 78 downregulated genes, were identified. The enriched functions and pathways of the DEGs include inflammatory and immune response, chemotaxis, extracellular matrix (ECM) organization, complement and coagulation cascades, ECM receptor interaction, and focal adhesion. The most significant module in the PPI network was analyzed and genes among it were mainly enriched in chemotaxis, locomotory behavior, immune response, chemokine signaling pathway, and extracellular space. In addition, DEGs, with degrees ≥ 10 and the top 10 highest Maximal Chique Centrality (MCC) score, were identified as hub genes. CCR1, MMP9, VCAM1, and ITGAX, which were of the highest degree or MCC score, were manually reviewed.

The DEGs and hub genes identified in the present study help us understand the molecular mechanisms underlying the pathogenesis of CAVD and might serve as candidate therapeutic targets for CAVD.

## 1 Introduction

Calcific aortic valve disease (CAVD) is the most common type of valvular disease and the primary cause of aortic stenosis in the world, which affects 2% to 7% of the population aged 65 years or older.^[1]^ CAVD presents with pathological thickening and calcification of the aortic valve (AV) and ultimately leads to its malfunction. It is believed that the disease burden of CAVD will double over the next 50 years which makes it an enormous threat to public health in the aging world.^[2]^ Despite that clinical features of CAVD have been established, the molecular basis remains unclear. Following the failures of statins and angiotensin-converting enzyme inhibitors in reducing CAVD progression,^[3–6]^ effective medical therapies are lacking. The only treatment option is surgical or transcatheter aortic valve replacement (TAVR) after CAVD develops into advanced stage and patients develop symptoms. However, these procedures are associated with high cost, inevitable risk of death, and perioperative as well as long-term complications such as complications accompanied by anticoagulation therapy and reoperation due to prosthetic valve dysfunction. There is, hence, an unmet clinical need for a better understanding of underlying mechanisms of CAVD and novel therapeutic targets to slow its progression. Thus, identification of critical genes, biomarkers, and pathways is critically important for early diagnosis, prevention, and precise treatment.

During the last decades, the high-throughput platforms for the analysis of gene expression, such as microarray technology, have been widely used with increasing value in screening genetic alterations at the genome level, which have helped us identify the differentially expressed genes (DEGs), functions, and pathways involved in the pathogenesis and progression of diseases. However, the small sample size of single microarray profile makes it difficult to obtain accurate results. Integrated bioinformatics analysis by using publicly available genomic data offers us possibilities for second data mining and identification of disease-related biomarkers.^[7,8]^ Thus, in the present study, 3 microarray datasets, GSE12644,^[9]^ GSE51472,^[10]^ and GSE83453^[11]^ were obtained from the NCBI-Gene Expression Omnibus database, which contained a total of 36 samples, with 20 cases of calcific aortic valves (CAVs) and 16 cases of normal AVs. We identified DEGs by using the R software with *Limma* package between CAVs and normal AVs. Gene ontology (GO), Kyoto Encyclopedia of Genes and Genomes (KEGG) pathway enrichment analysis was performed, and protein–protein interaction (PPI) network was constructed to explore the molecular mechanisms underlying CAVD. Subsequently, we screened the most significant module and hub genes in the PPI network established by DEGs. Publications related to the hub genes, functions, and pathways revealed by the above analysis were manually reviewed and were discussed in the Discussion section. Our study provides potential targets for treating CAVD.

## 2 Material and methods

### 2.1 Microarray data

Gene Expression Omnibus Database (GEO) (http://www.ncbi.nlm.nih.gov/geo)^[12]^ is a public functional genomics data repository of high throughout gene expression data, chips, and microarrays. Three gene expression datasets GSE12644,^[9]^ GSE51472,^[10]^ and GSE83453^[11]^ were downloaded from GEO. All the microarray data of GSE12644, GSE51472, and GSE83453 were based on GPL570 platform (Affymetrix Human Genome U133 Plus 2.0 Array) and include a total of 20, 15, and 15 samples of AVs, respectively. Only the samples of CAVs and normal AVs were taken into analysis which constituted a total of 36 samples (20 CAVs and 16 normal aortic valves).

### 2.2 Identification of DEGs

The downloaded platform and series of matrix files were converted by using the R software. The DEGs between CAVs and normal AVs were screened by using *Limma* package in the R software. An adjusted *P* value < .05 and | Fold Change (FC) |≥ 2 were set as cut-off criteria at first. However, there were no enough DEGs identified in GSE83453 for further analysis. To screen enough DEGs for a better identification of the underlying critical genes, the | FC | cutoff of GSE83453 was set as ≥ 1.5^[13]^ individually while all the other parameters among 3 datasets remained unchanged. The DEG data were processed to draw heatmaps of the top 500 significantly changed genes by using *Gplots* package in the R software. All codes were run under the R environment version 3.5.3.

### 2.3 GO and KEGG pathway enrichment analysis of DEGs

The Database for Annotation, Visualization and Integrated Discovery (DAVID; http://david.ncifcrf.gov) (version 6.8)^[14]^ was used to provide a comprehensive set of functional annotation information of genes and proteins. GO is an important bioinformatics tool to annotate and illustrate genes and their biological process (BP), cellular component (CC), and molecular function (MF).^[15]^ KEGG is a comprehensive database resource, which contains information of high-level functions and biological systems from large-scale molecular datasets.^[16]^ GO and KEGG enrichment analysis of DEGs were performed by using DAVID online database. *P* value < .05 was considered statistically significant.

### 2.4 PPI network construction and module analysis

The PPI network of DEGs was constructed by using Search Tool for the Retrieval of Interacting Genes (STRING; http://string-db.org) (version 11.0)^[17]^ database, and an interaction with a combined score > 0.4 was considered statistically significant. Cytoscape (version 3.7.1) is an open-source bioinformatics software platform for visualizing molecular interaction networks.^[18]^ The plugin Molecular Complex Detection (MCODE) (version 1.5.1) is an application for clustering a given network based on topology to find densely connected regions.^[19]^ The PPI networks were imported into Cytoscape and the most significant module in the PPI networks was identified by MCODE. The criteria for selection were as follows: MCODE scores > 5, degree cutoff = 2, node score cutoff = 0.2, max depth = 100, and k-score = 2. GO and KEGG analyses for genes in this module were performed by using DAVID.

### 2.5 Hub genes identification and analysis

The Cytoscape plugin *cytoHubba* is an application for ranking nodes in a network by their network features. The hub genes were calculated based on the Maximal Chique Centrality (MCC) topological analysis methods^[20]^ by using *cytoHubba* (version 0.1).

## 3 Results

### 3.1 Identification of DEGs in CAVD

**Figure 1:**
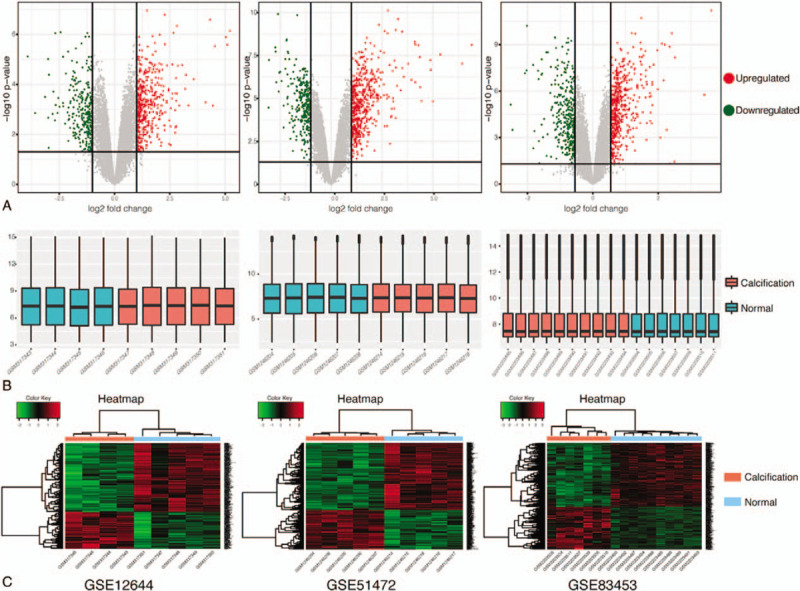
Data standardization and DEG identification in 3 microarray datasets (GSE12644, GSE51472, and GSE83453). A, Respective volcano plots of the 3 datasets. Red plots represent the upregulated genes and green ones represent the downregulated genes with the criteria of *P* value < .05 and | FC |≥ 2 in GSE12644 and GSE51472 or | FC |≥ 1.5 in GSE83453. B, Poststandardization gene expression levels of each dataset. C, Heatmap of the top 500 significant DEGs. Red and green indicate higher and lower gene expression, respectively. DEG = differentially expressed gene, FC = fold change.

**Figure 2:**
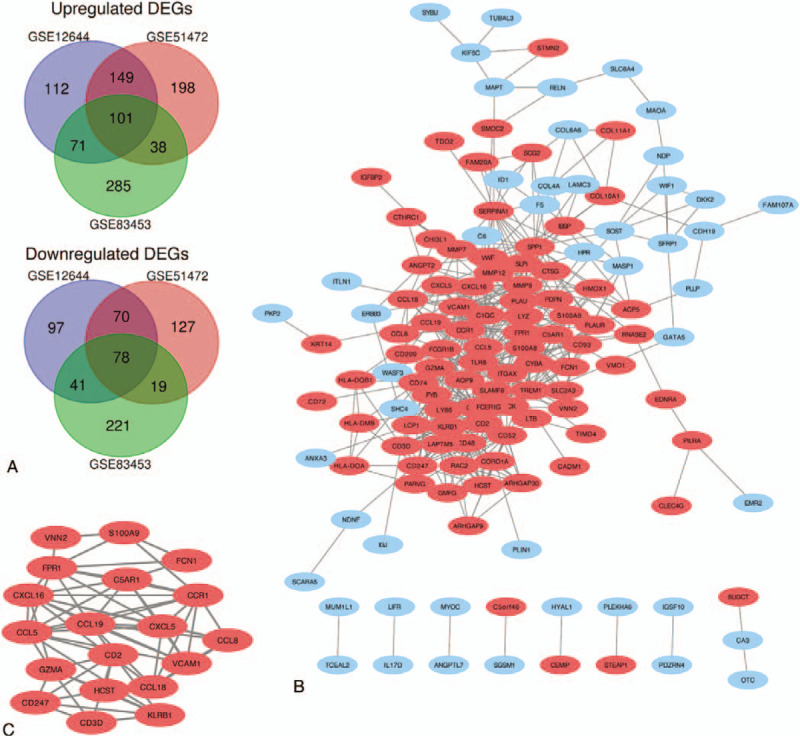
Venn diagram, PPI network, and the most significant module of DEGs. A, DEGs identified from the 3 microarray datasets showed overlaps of 101 upregulated DEGs and 78 downregulated DEGs. The upregulated genes are marked in red and downregulated genes are marked in blue. B, The PPI network of DEGs was constructed using Cytoscape. C, The most significant module was obtained from PPI network with 19 nodes and 64 edges. DEG = differentially expressed gene, PPI = protein–protein interaction.

A total of 36 samples (20 CAVs and 16 normal aortic valves) were selected. All the tissues, except tissues from GSE51472 which did not provide baseline information, were from male patients and there was no significant difference in patient's age (64.53 ± 5.00 vs 60.82 ± 5.40, *P* = .08, CAV group vs normal AV group). We respectively identified 719, 780, and 854 DEGs from GSE12644, GSE51472, and GSE83453 (Fig. 1A) after standardization of the microarray datasets (Fig. 1B). The overlap among the 3 datasets contained 101 upregulated genes and 78 downregulated genes between CAVs and normal AVs (Fig. 2A). The top 500 significant DEGs were depicted as a heatmap (Fig. 1C).

### 3.2 GO and KEGG pathway enrichment analysis of DEGs

**Figure 3:**
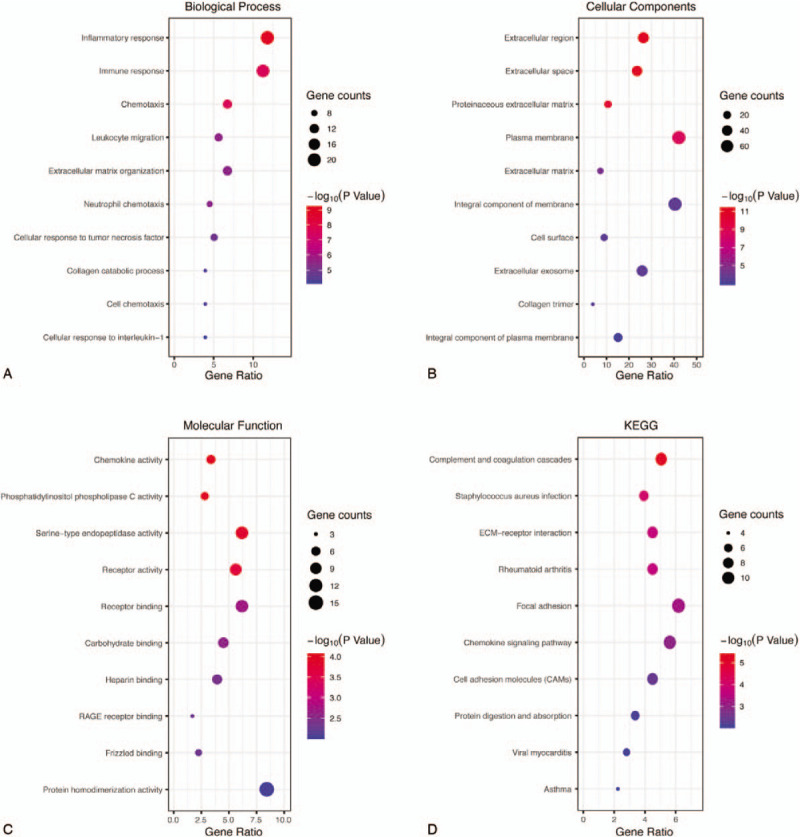
GO and KEGG pathway enrichment analysis of DEGs. A, The top 10 most significantly enriched GO terms of DEGs in biological process analysis. B, The top 10 most significantly enriched GO terms of DEGs in cellular component analysis. C, The top 10 most significantly enriched GO terms of DEGs in molecular function analysis. D, The top 10 most significantly enriched pathways of DEGs in KEGG pathway analysis. DEG = differentially expressed gene, GO = gene ontology, KEGG = Kyoto Encyclopedia of Genes and Genomes.

The function and pathway enrichment analysis of DEGs was performed by using DAVID. GO enrichment analysis showed that the changes of DEGs in BP were most significantly enriched in inflammatory response, immune response, chemotaxis, leukocyte migration, and extracellular matrix (ECM) organization (Table 1 and Fig. 3A). Changes of DEGs in CC were most significantly enriched in extracellular region, space, and plasma membrane (Table 1 and Fig. 3B). Changes of DEGs in MF were most significantly enriched in chemokine activity, phosphatidylinositol phospholipase C activity, serine-type endopeptidase activity, receptor activity, and receptor binding (Table 1 and Fig. 3C). KEGG pathways analysis revealed that DEGs were mainly enriched in complement and coagulation cascades, staphylococcus aureus infection, ECM–receptor interaction, rheumatoid arthritis (RA), and focal adhesion (Table 2 and Fig. 3D).

**Table 1:**
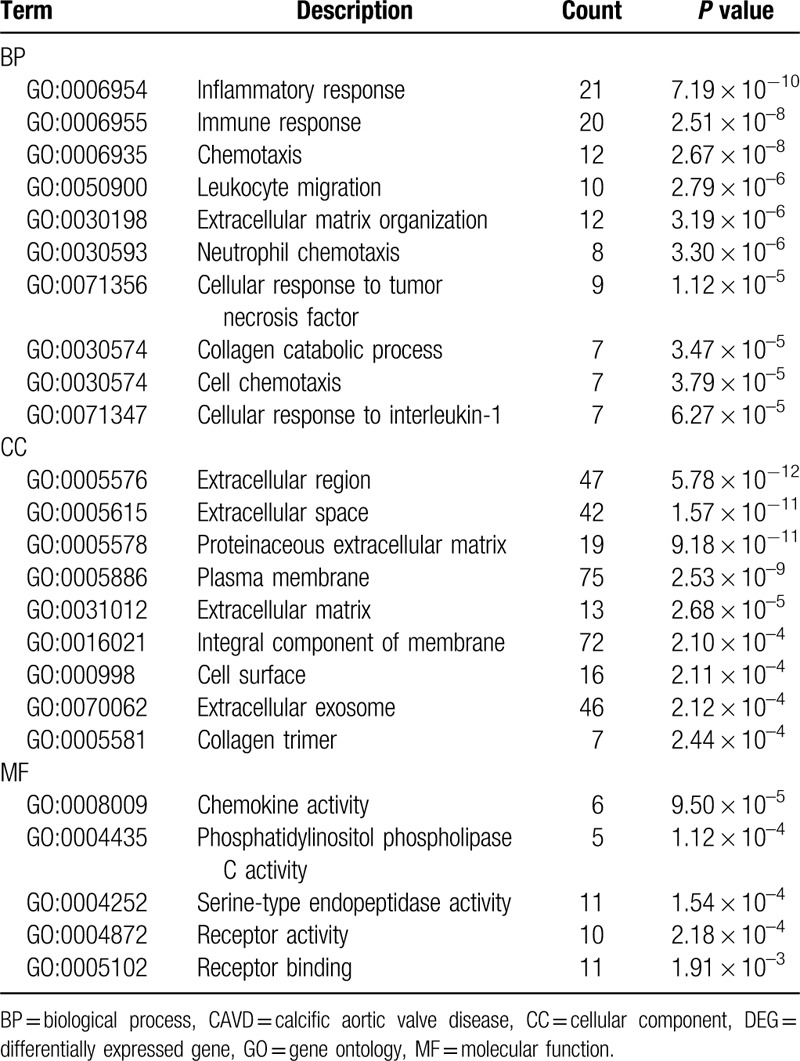
GO enrichment analysis of DEGs in CAVD.

**Table 2:**
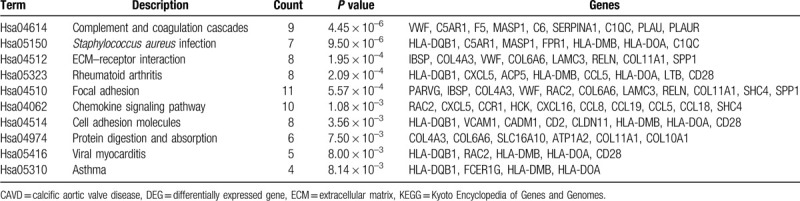
KEGG pathway enrichment analysis of DEGs in CAVD.

### 3.3 PPI network construction, module analysis

The PPI network was constructed and contained 178 nodes and 519 edges (Fig. 2B). The most significant module consisted of 19 nodes and 64 edges, was calculated and identified by using Cytoscape plugin MCODE (Fig. 2C). The function and pathway enrichment analysis of genes involved in this module were analyzed by using DAVID. Results showed that genes in this module were mainly enriched in chemotaxis, locomotory behavior, immune response, chemokine signaling pathway, and extracellular space (Table 3).

**Table 3:**
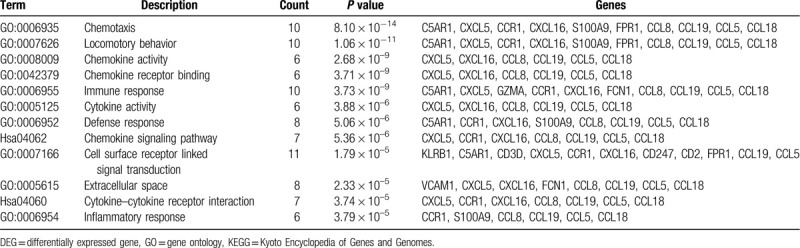
GO and KEGG pathway enrichment analysis of DEGs in the most significant module.

### 3.4 Hub genes identification and analysis

A total of 44 genes were identified with degrees ≥ 10 and they were subsequently calculated based on the MCC analysis method, which is the latest analysis method and is highly recommended. The top 10 genes with highest MCC score were considered as hub genes (Table 4).

**Table 4:**
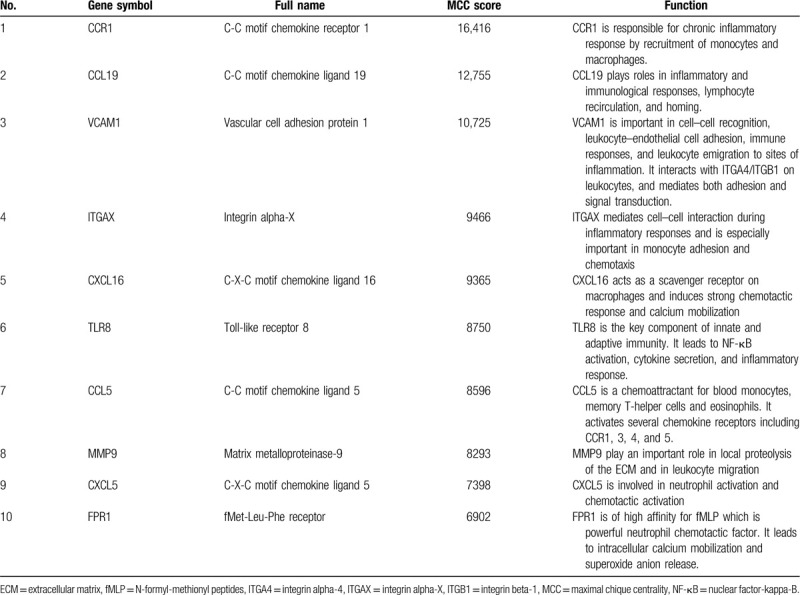
Functional roles of top 10 hub genes with highest MCC score.

## 4 DISCUSSION

CAVD is a progressive and irreversible disease that remains incurable despite dramatic improvement in surgical treatment. In this study, we analyzed the expression of genes in 3 microarray datasets between CAVs and normal AVs. A total of 179 DEGs were identified, including 101 upregulated genes and 78 downregulated genes. Subsequently, we utilized bioinformatic methods to deeply explore the DEGs, including GO and KEGG pathway enrichment analysis, PPI network construction, identification of the most significant module, and the hub genes.

The GO and KEGG pathway analyses were performed to explore interactions among the DEGs. GO enrichment analysis contains 3 groups: BP, CC, and MF. For BP, DEGs were mainly involved in inflammatory and immune responses, chemotaxis, leukocyte migration, and ECM organization. CAVD is believed as a chronic inflammatory disease.^[21,22]^ Endothelial cells on aortic valve surface interact with aortic valve interstitial cells (AVIC) to maintain the integrity of valve tissues. Endothelial dysfunction and injury lead to the impaired endothelial integrity and thus cause inflammatory responses including lipid deposition, intraleaflet hemorrhage, and oxidative stress. Subsequent innate and adaptive immune responses^[23,24]^ are activated through chemotaxis^[25]^ and extensive aortic valve infiltration with macrophages, T cells, and mast cells.^[26–28]^ In addition, ECM organization is one of the hallmarks of CAVD which have been reported previously.^[29,30]^ CAV is characterized by fibrotic thickening of the valve leaflets, inflammation, neoangiogenesis, calcification, and the presence of other ectopic mesenchymal tissues, especially in the fibrosa layer.^[31]^

For CC, DEGs were mainly enriched in extracellular region and space, which is consistent with the above-mentioned ECM organization. For MF, DEGs were mainly enriched in chemokine activity, phosphatidylinositol phospholipase C (PI-PLC) activity, serine-type endopeptidase activity, receptor activity, and binding. Chemokine is a group of low molecular weight cytokines with the ability to induce chemotaxis or chemokinesis in leukocytes, which plays a critical role in inflammatory response. Recent studies have elucidated partial mechanisms between chemokines and CAVD.^[32,33]^ PI-PLC pathway, which is the predominant Ca^2+^ release mechanism in smooth muscle cells and nonexcitable cells (e.g., epithelial cells and fibroblasts),^[34,35]^ has been reported playing a key role in smooth muscle cell proliferation and vascular calcification.^[36]^ High-temperature requirement serine protease A1 is a member of the trypsin family of serine-type endopeptidase, and has been elucidated of its important role in cell growth regulation and the strong relations with cartilage ossification in osteoarthritis.^[37]^

Following KEGG pathway enrichment analysis showed that DEGs were mainly enriched in complement and coagulation cascades, staphylococcus aureus infection, ECM–receptor interaction, RA, and focal adhesion. Complement and coagulation cascades evolutionarily related proteolytic cascades associated with inflammatory response.^[38]^ Coagulation cascade, initiated by tissue factor, has been reported to play an important role in mineralization process of aortic valve by cleaving osteopontin, which is an important regulator of calcium deposition in bone and ectopic calcified tissue.^[39]^ Complement cascade regulates the recruitment of inflammatory and immunocompetent cells and is involved in the structural deterioration of heart valves.^[40]^ KEGG pathway enrichment in staphylococcus aureus infection is due to the common activated pathways or pathological processes both in the CAVD and staphylococcus aureus infection, which include neutrophil chemotaxis, complement activation, and secretion of immune modulating proteins.^[41]^ ECM interaction between cells and ECM regulates cellular activities such as focal adhesion, migration, AVICs transformation.^[42,43]^ In patients with RA, abnormal activation of immune response leads to the elevated level of pro-inflammatory cytokines and chemokines, which promotes leukocyte infiltration. It is well recognized that there are strong relationships and common underlying mechanisms shared between RA and aortic valve stenosis.^[44,45]^ In a word, all these theories are consistent with our enrichment analysis results.

A PPI network of DEGs were constructed, containing 178 nodes and 519 edges with an average node degree of 5.83. Among the network, only 1 significant module with MCODE score > 5 was identified based on the degree of importance. It contained 19 nodes and 64 edges. The GO enrichment analysis revealed that DEGs in this module were mainly enriched in chemotaxis, locomotory behavior, chemokine activity, immune response, and cytokine activity, while in KEGG were mainly enriched in chemokine signaling pathway and cytokine–cytokine receptor interaction.

There were a total of 44 DEGs with degrees ≥ 10. So, we applied MCC method, the latest and the most recommended analysis method, for further screening of hub genes. The top 10 DEGs with highest MCC score were considered hub genes in our study. Among these hub genes, CCR1 showed the highest MCC score and matrix metalloproteinase-9 (MMP9) showed the highest node degree, respectively, while VCAM1 and integrin alpha-X (ITGAX) were both ranked top 5 in both the lists according to the above-mentioned 2 analysis methods.

CCRs (C-C motif chemokine receptors) family, including CCR1, plays an important role in chronic inflammatory response by recruitment of monocytes and macrophages.^[46]^ Although without CCR1, increasing evidences indicate that CCRs are also involved in cardiovascular calcification. For instance, CCR5 was revealed with positive correlation with the valvular calcification^[33]^ and CCR2 was confirmed with the ability of osteoblastic transformation of valvular interstitial cells.^[32]^ Hence, the function of CCR1 in CAVD is worth to be further studied. MMP9 participates in the degradation and reorganization of ECM, which is one of the most histopathological features of the valvular disease.^[47,48]^ It has been identified as a novel predictor of cardiovascular events,^[49]^ as well as left ventricular dilation.^[50]^ Even in a clinical trial, MMP9 showed increased risk trend of combined endpoints at 1 year after TAVR procedure (*P* value = .086, 95% confident interval 0.523–1.000).^[51]^ To some degree, MMP9 is highly correlated with CAVD^[52,53]^ and is generally regarded as the biomarker of CAVD and heart remodeling.^[54]^ Vascular cell adhesion molecule 1 (VCAM1) mediates leukocyte–endothelial cell adhesion and has been consistently reported with increased level in endothelium of CAV.^[55–57]^ The endothelial cells of aortic valve, activated by the altered hemodynamics stimuli,^[55]^ expressing VCAM1 and other adhesion molecules, lead to the main route for tissue leukocyte infiltration and inflammatory process. Moreover, circulation VCAM1 is reported to be associated with greater coronary artery disease risk in chronic inflammatory conditions.^[58]^ ITGAX, also known as CD11c, is highly expressed in CD11c-positive (M1) macrophages which involved in inflammatory response.^[59]^ The shift of macrophage toward higher proportional CD11c-positive phenotype promotes the osteogenic differentiation of AVICs and aortic valve calcification.^[59]^

CAVD has long been considered to be resulted from a degenerative process and involved in pathogenesis with many similarities to atherosclerosis. The disruption of valve endothelium due to high shear stress and subsequent lipid deposition lead to the aortic valve lesions. Hence, the anti-inflammatory and anticalcific effects of statins have been taken into consideration and clinical trials were performed. However, 3 large prospective double-blinded randomized placebo-controlled trials of statins in CAVD failed to show any retardation of its progression.^[4–6]^ The similar result was obtained from the clinical trial of angiotensin-converting enzyme inhibitors.^[3]^ In our study, the CCR1, which had the highest MCC score, was identified as hub genes in CAVD. It is also found that CCR1 plays an important role in inflammatory conditions such as RA through chemotaxis, which is consistent with our enrichment analysis result. Several oral CCR1 antagonists had been developed for immunologically mediated inflammatory diseases such as RA, multiple sclerosis, and chronic obstructive pulmonary disease.^[60–62]^ CCX354-C is a small molecule CCR1 antagonist that inhibits CCR1-dependent signaling, including chemotaxis. CCR1 Antagonist Rheumatoid Arthritis Trial 2 (CARAT-2), a phase II double-blind, randomized, placebo-controlled clinical trial, found that CCX354-C is effective and tolerated in 160 patients with RA.^[63]^ Based on the CARAT-2 results, it is encouraging and promising that CCR1 antagonist might be a potential therapeutic target for CAVD treatment, which should be further studied and solved.

## 5 Conclusions

The present study performed integrated bioinformatics analysis by using microarray datasets of CAVs and normal aortic valves. A total of 179 DEGs and 10 hub genes were identified, which mainly enriched in inflammatory and immune responses. These genes and pathways might be potential therapeutic targets for CAVD. Moreover, CCR1 might be a promising one due to its effectiveness and safety in RA treatment, which shares partial common underlying mechanisms with CAVD. This study increased our understanding of the molecular drivers that underlie CAVD and further studies are needed to elucidate the biological function of these genes in CAVD.

## Author contributions

Peng Teng and Xingjie Xu performed the bioinformatics analysis. Haimeng Yan and Qianhui Sun mined and downloaded the data from the GEO database. Chengyao Ni analyzed and interpreted the data. Enfan Zhang and Yiming Ni provided methodological guidance.
